# Sacro-Peptones

**Published:** 1885-02

**Authors:** Joseph A. Treat


### SACRO-PEPTONES.

In a singularly distressing case, which has been under my observation, I have observed remarkable results from the use of sacro-peptones. The case was one of neuralgic dyspepsia. The patient was unable to assume a recumbent position, and was troubled with constant eructation of gas. He was literally starving to death, through inability to retain anything on his stomach for longer than from one to three minutes. He had tried the various foods and peptonized preparations on the market, without avail. After putting him on the sacro-peptones he rejected a portion of the first three or four doses administered. But after this his stomach became quiet and he retained the preparation without difficulty. Although he has now been taking sacro-peptones continuously for a considerable length of time, they have not become distasteful, as is to apt to be the case in most other forms of peptonized beef. The case is a chronic one, its duration extending back ten or twelve years, which fact makes the success of the sacro-peptones all the more remarkable.—*Joseph A. Treat, M. D., in Therapeutic Gazette.*

